# Is There an Association between Bell Palsy in Pediatric Patients and COVID-19?

**DOI:** 10.1055/s-0044-1789197

**Published:** 2025-01-23

**Authors:** Mohamed E. El-Deeb, Saad Elzayat, Abeer Salamah, Ali Gamal, Shimaa Elgamal, Ahmed El-Sobki

**Affiliations:** 1 Department of Otorhinolaryngology, Head and Neck Surgery, Kafrelsheikh University Hospital, Faculty of Medicine, Kafrelsheikh University, Kafr El Sheikh, Kafr El Sheikh Governorate, Egypt; 2 Department of Pediatrics, Faculty of Medicine, Kafrelsheikh University, Kafr El Sheikh, Kafr El Sheikh Governorate, Egypt; 3 Department of Neuropsychiatry, Faculty of Medicine, Kafrelsheikh University, Kafr El Sheikh, Kafr El Sheikh Governorate, Egypt; 4 Department of Otorhinolaryngology, Faculty of Medicine, Mansoura University, Mansoura, Dakahlia Governorate, Egypt

**Keywords:** Bell palsy, function recovery, COVID-19, facial nerve

## Abstract

**Introduction** Bell palsy (BP) is an acquired, idiopathic facial palsy linked to lower motor neuron malfunction of the seventh cranial nerve. Several studies have identified BP as one of the many neuropathies that coronavirus disease 2019 (COVID-19) patients have developed, while other studies disagree.

**Objective** To study if there is an association between BP in pediatric patients and COVID-19, and to examine the pattern of recovery in all pediatric cases of BP during the COVID-19 pandemic.

**Methods** We performed a prospective cohort study on pediatric patients with acute onset unilateral facial weakness of unknown etiology (BP) during the pandemic period. All included patients were submitted to a reverse transcription-polymerase chain reaction (RT-PCR) test through nasopharyngeal and oropharyngeal swabs for severe acute respiratory syndrome coronavirus 2 (SARS-CoV-2) at the time of the BP diagnosis.

**Results** No significant differences were found regarding COVID-19 infection and recovery from BP at the first, third, or sixth months of follow-up. According to the results, it seems that there is no association between COVID-19 infection and facial palsy; however, the patients infected with COVID-19 in the sample experienced a rapid, early recovery from BP. The mean incidence of BP in 5 years (2017–2021) was of 1.73/100 thousand individuals, with a statistically insignificant change throughout the years.

**Conclusion** We were not able to show any association between BP and COVID-19. The patients underwent follow-up for up to 6 months, and we studied their patterns of recovery from BP, which were like those observed before the pandemic.

## Introduction

Bell palsy (BP) is an acquired, idiopathic facial palsy linked to lower motor neuron malfunction of the seventh cranial nerve. It typically affects one side of the face and is characterized by weakening or palsy of the facial muscles. 1

When no other reason can be found, BP is diagnosed through exclusion criteria, and inflammatory, traumatic, and viral processes are the most frequently acknowledged causes. The condition is associated with viral infection, 2 usually by members of the *Herpesviridae* family.

Facial nerve palsy (FNP) is a condition that frequently manifests in emergency rooms in pediatric patients who have suffered accidents. Because of its abrupt onset and frequently rapid progression, it needs to be diagnosed as soon as possible to minimize the effects on patient morbidity and quality of life. 3

A frequent issue, FNP significantly lowers the quality of life of patients. The incidence rates among children are of 2.7 per 100 thousand individuals per year for those under the age of 10, and of 10.1 per 100 thousand individuals per year for those between the ages of 10 and 20. Yet, the prevalence rate varies from 10 to 30 per 100 thousand individuals. In the pediatric emergency room, FNP is a concerning symptom that demands an immediate diagnosis. 4

In children, BP has a favorable prognosis, with studies 5 indicating that 80 to 90% of children recover fully within 6 months of symptom onset.

Severe acute respiratory syndrome coronavirus 2 (SARS-CoV-2) has been identified as a highly-morbid and potentially-fatal virus that causes coronavirus disease 2019 (COVID-19), which developed into a pandemic. It was isolated from human airway epithelial cells and belongs to the coronavirus family. With an average incubation period of 5 to 7 days, this virus spreads by droplets and direct contact. 6 7

It is known that coronaviruses have a propensity for neuroinvasiveness, 8 and COVID-19 patients may exhibit neurological symptoms. When COVID-19 initially manifests, neurological symptoms, such as headaches, hyposmia, hypogeusia, dizziness, disorientation, cerebrovascular disorders, Guillain-Barré syndrome (GBS), and encephalopathies may also occur concurrently with respiratory symptoms. Up to 36% of COVID-19 patients have exhibited neurological symptoms, particularly those with severe respiratory tract infections. 9 10

One of the several neuropathies that COVID-19 patients have experienced, in many cases BP was the only significant neurological condition documented. 11 Several studies have identified FNP because of COVID-19, either as an isolated symptom in otherwise asymptomatic patients or in combination with other COVID-19 symptoms. 12

The present work aims to study if there is an association between BP and COVID-19 in pediatric patients, as well as the pattern of recovery in pediatric cases of BP during the COVID-19 pandemic.

## Methods

We performed a prospective cohort study on pediatric patients with acute onset unilateral facial weakness of unknown etiology (BP) during the pandemic period.

The study was conducted at the Department of Otorhinolaryngology and Head and Neck Surgery, Kafrelsheikh University Hospital, from July 2020 to June 2022 and was approved by the hospital's Ethics Committee (under code MKSU 50–2-7). Written informed consent was obtained from the parents of the included patients.

All patients in the study presented to our clinic with a complaint of acute facial paralysis; as part of the routine process to investigate the etiology of the facial paralysis, they were submitted to a detailed anamnesis, an ear, nose, and throat examination, a complete neurological examination, complete blood count, biochemical blood tests, and viral serologies (particularly herpes simplex virus [HSV], human immunodeficiency virus [HIV], and varicella-zoster virus [VZV]); a history of trauma warrants investigation for other stigmata of temporal bone fractures during the physical examination and cranial and temporal MRI scans were also performed. We excluded adult patients (older than 18 years) and those submitted to immunization with a live vaccine or COVID-19 vaccine within the previous month.

After ruling out the known causes, patients with idiopathic lower motor neuron facial paralysis (BP) were included in the present study. Furthermore, for all included patients, we performed a reverse transcription-polymerase chain reaction (RT-PCR) test through nasopharyngeal and oropharyngeal swabs for SARS-CoV-2 at the time of the BP diagnosis. The severity of the FNP was assessed and graded using the House-Brackmann (H-B) score. 13

All patients underwent the same treatment protocol (except if there was a contraindication to corticosteroids), which included prednisone 1 mg/kg/day, which was tapered and stopped in 2 weeks, in addition to meticulous eye care. All patients were followed up at 1 week, 2 weeks, 1 month, 3 months, and 6 months to check for resolution of the facial weakness.

The sample was divided into three groups according to the degree of recovery by comparing the severity of BP between the time of diagnosis and the patient's follow-up visit: complete recovery, partial recovery, and non-recovery. The complete recovery group was defined as H-B grade 1, the partial recovery group was defined as those who had experienced an improvement of at least 1 H-B grade, but not an improvement to grade 1, and the non-recovery group was composed of the subjects whose severity score had not changed or was worse than when they had been diagnosed.

The participants who had fully recovered at the 1-month follow-up visit were not required to return to the study site for the remaining time points of the study; instead, their parents/guardians were asked by phone about the child's condition and, in case of the appearance of new manifestations, they were asked to visit our clinic. Similarly, participants with full recovery at the 3-month follow-up visit were not required to return to the study site for the follow-up at 6 months.

We also calculated the incidence rate of pediatric cases of BP in the past 5 years in the Kafr El Sheikh Governorate, where the study was conducted. The number of new BP cases admitted to our hospital in that period was divided by the pediatric population (individuals younger than 18 years) of the area to calculate the yearly incidence rates, which were compared with those of other years.

### Statistical Analysis

The data was analyzed using the IBM SPSS Statistics for Windows (IBM Corp., Armonk, NY, United States) software, version 26.0. The quantitative variables were expressed as mean and standard deviation values, and the categorical variables, as absolute frequencies; the Chi-squared trend test was used to compare them (in the case of ordinal data). To validate the assumptions for use in parametric tests, the Kolmogorov-Smirnov (distribution-type) and Levene (homogeneity of variances) tests were used. The Wilcoxon signed-rank test was used to compare one group in terms of an ordinal variable throughout two time points. To investigate differences in variable distribution within one group, the one-sample Chi-squared test was used. The level of statistical significance was set at *p* < 0.05. A highly significant difference was present if *p* ≤ 0.001.

## Results

The present study included 27 patients with facial palsy, 15 (55.6%) of whom were female subjects. The age ranged from 2 to 16 (mean: 9.04 ± 3.14) years. In total, 14 patients presented a left-side lesion. There were only 2 (7.4%) cases of confirmed COVID-19 infection, with a statistically significant difference in terms of distribution ( Table 1 ).

**Table 1:** Distribution of patients (N = 27) according to demographic data

	n	%
**Gender**		
Female	15	55.60%
Male	12	44.40%
**Age (years):**		
Mean ± standard deviation	9.04 ± 3.14	
Range	−16	
**Side of the lesion**		
Left	14	51.90%
Right	13	48.10%
**SARS-COV-2 RT PCR:**		
Negative	25	92.60%
Positive	2	7.40%
***p***	< 0.001**	

**Abbreviations:** RT-PCR, reverse transcription-polymerase chain reaction; SARS-CoV-2, severe acute respiratory syndrome coronavirus 2.

**Notes:** **One-sample Chi-squared test; statistically, *p* ≤ 0.001 is highly significant in the assessment of the grade of facial nerve affection in coronavirus disease 2019 (COVID-19) patients; the only 2 patients with confirmed COVID-19 in the present study were grade IV on the House-Brackman facial nerve grading system.

After 1 month, 15 patients (55.6%) were H-B grade I, and there were no cases of grade V with significant improvement ( *p* < 0.001). After 3 months, 20 patients (74.1%) were H-B grade I, with significant improvement at 3 months when compared with the evaluation after one month. The rate of grade-I patients rose to 81.5% after 6 months, without significant changes when compared with the assessment after 3 months ( Table 2 ).

**Table 2:** Change in House-Brackman grade throughout 6 months among the studied patients (N= 27)

House-Brackmann grade	Initial: n (%)	After 1 month: n (%)	After 3 months: n (%)	After 6 months: n (%)
I II III IV V	0 (0%) 3 (11.1%) 8 (29.6%) 13 (48.1%) 3 (11.1%)	15 (55.6%) 6 (22.2%) 2 (7.4%) 7 (14.8%) 0 (0%)	20 (74.1%) 2 (7.4%) 3 (11.1%) 2 (7.4%) 0 (0%)	22 (81.5%) 1 (3.7%) 2 (7.4%) 2 (7.4%) 0 (0%)
*p*		< 0.001**	0.011*	0.083

**Notes:** the *p* -values pertain to the Wilcoxon signed-rank test; *statistically significant; ** highly significant.

After 1 month, 15 patients (55.6%) presented complete improvement, while 3 patients (11.1%) presented no improvement. In the third month, 20 patients (74.1%) were grade I, with significant improvement when compared with the evaluation after 1 month. After 6 months, no significant changes were observed compared with assessment after 3 months, in which 22 patients (81.5%) showed complete improvement ( Table 3 ).

**Table 3:** Recovery of the facial nerve throughout 6 months among the studied patients (N = 27)

Recovery	After 1 month: n (%)	After 3 months: n (%)	After 6 months: n (%)
Complete Partial None	15 (55.6%) 9 (33.3%) 3 (11.1%)	20 (74.1%) 5 (18.5%) 2 (7.4%)	22 (81.5%) 3 (11.1%) 2 (7.4%)
*p*		0.014*	0.157

Notes: the *p* -values pertain to the Wilcoxon signed-rank test; *statistically significant.

No significant differences were found regarding gender and recovery in the first, third, or sixth months. By the end, 2 female patients (7.4%) presented no improvement, and all male patients presented some degree of improvement. Complete improvement was observed in 11 (73.3%) of the female patients and in 11 (91.7%) of the male patients ( Table 4 ).

**Table 4:** Relationship between gender and recovery from facial nerve affection

Recovery	Female patients (N = 15)	Male patients (N = 12)	χ ^2^	*p*
	n (%)	n (%)		
**After 1 month**			0.855	0.355
Complete	8 (53.3%)	7 (58.3%)		
Partial	4 (26.7%)	5 (41.7%)		
None	3 (20%)	0 (0%)		
**After 3 months**			3.51	0.061
Complete	9 (60%)	11 (91.7%)		
Partial	4 (26.7%)	1 (8.3%)		
None	2 (13.3%)	0 (0%)		
**After 6 months:**			1.892	0.169
Complete	11 (73.3%)	11 (91.7%)		
Partial	2 (13.3%)	1 (8.3%)		
None	2 (13.3%)	0 (0%)		

**Abbreviation:** χ ^2^ , Chi-squared trend test.

There was a statistically insignificant relationship between age and recovery in the first, third, or sixth months. By the end of 6 months, all patients aged < 9 years presented complete resolution, as well as 12 (70.6%) of those aged ≥ 9 years ( Table 5 ).

**Table 5:** Relationship between age and recovery from facial nerve affection

Recovery	< 9 years (N = 10)	≥ 9 years (N = 17)	χ ^2^	*p*
	n (%)	n (%)		
**After 1 month:**			0.064	0.8
Complete	5 (50%)	10 (58.8%)		
Partial	4 (40%)	5 (29.4%)		
None	1 (10%)	2 (11.8%)		
**After 3 months**			2.248	0.134
Complete	9 (90%)	11 (64.7%)		
Partial	1 (10%)	4 (23.5%)		
None	0 (0%)	2 (11.8%)		
**After 6 months:**			3.022	0.082
Complete	10 (100%)	12 (70.6%)		
Partial	0 (0%)	3 (17.6%)		
None	0 (0%)	2 (11.8%)		

**Abbreviation:** χ ^2^ , Chi-squared trend test.

No significant differences were found involving COVID-19 infection and recovery in the first, third, or sixth months. A total of 2 patients (7.4%) had COVID-19 infection and experienced complete improvement in the first month, as did 13 (52%) of non-COVID patients. According to the results, it seems that there is no association between COVID-19 infection and facial palsy; however, the patients infected with COVID-19 in the sample experienced a rapid, early recovery from BP ( Table 6 ).

**Table 6:** Relationship between COVID-19 infection and recovery from facial nerve affection

Recovery	Negative patients (N = 25)	Positive patients (N = 2)	χ ^2^	*p*
	n (%)	n (%)		
**After 1 month**			1.368	0.242
Complete	13 (52%)	2 (100%)		
Partial	9 (36%)	0 (0%)		
None	3 (12%)	0 (0%)		
**After 3 months**			0.624	0.43
Complete	18 (72%)	2 (100%)		
Partial	5 (20%)	0 (0%)		
None	2 (8%)	0 (0%)		
**After 6 months:**			0.411	0.521
Complete	20 (80%)	2 (100%)		
Partial	3 (12%)	0 (0%)		
None	2 (8%)	0 (0%)		

**Abbreviation:** χ ^2^ , Chi-squared trend test; COVID-19, coronavirus disease 2019.

The mean incidence of BP in 5 years (2017–2021) was of 1.73/100 thousand individuals, with a statistically insignificant change throughout the years ( Table 7 ).

**Table 7:** Incidence rate of facial palsy from 2017 to 2021

Year	Cases	Pediatric population	Incidence rate	Mean incidence
2017	13	866,138	1.5 /100,000	1.73/100,000; *p* = 0.2
2018	16	883,929	1.81 /100,000	
2019	15	901,572	1.66 /100,000	
2020	16	919,052	1.74 /100,000	
2021	18	936,330	1.92 /100,000	

## Discussion

Although the precise pathophysiology of acute-onset facial nerve paralysis is unknown, it is believed to be connected to axonal spread and viral replication in conjunction with neurotropic herpes viruses (HSV and VZV), which cause inflammation and demyelination in the nerve. 14 In the population of children, BP accounts for 60 to 80% of cases of FNP. 15

The olfactory nerve and bulb, which are directly connected to the central nervous system, are two pathways by which SARS-CoV-2 might enter the central nervous system. The other is through viremia. To date, there are few reports in the literature of investigations on the neurological side effects of COVID-19, such as the study by Mao et al. 9

It is still unclear how SARS-CoV2 manifests in young patients. Pediatric patients have been observed to have respiratory conditions, leukopenia, thrombocytopenia, myocarditis, interstitial pneumonia, Kawasaki-like sickness, and vasculitis. 16

### Incidence Rate of Facial Nerve Palsy in Children

In the present study, the mean incidence of BP in children from 2017 to 2021 was of 1.73/100 thousand individuals, with a statistically insignificant change throughout the years.

The incidence of BP is debatable; however, Yılmaz et al., 17 in a retrospective investigation of 81 children (aged 1 to 16 years) in Turkey between 2011 and 2013, found that 80.2% of peripheral FNP cases had idiopathic causes. According to Rowhani-Rahbar et al., 18 for every 100 thousand children under the age of 18, there are 19 to 21 cases of BP. Rowlands et al. 19 have stated that FNP is expected to affect 2.7 per 100 thousand children under the age of 10, and 10.1 per 100 thousand children aged 10 to 20.

According to Mutlu et al., 20 98% of patients referred to their clinic with idiopathic lower motor neuron FNP received negative results from SARS-CoV-2 RT-PCR tests, and there was no rise in the number of patients with this condition during the COVID-19 outbreak.

The incidence of pediatric FNP in 2020 was not noticeably different from the rate in the preceding 5 years, according to Barron et al. 21 On the other hand, Zammit et al. 22 found a statistically significant increase in the number of cases of BP in 2020 (3.5%) compared with 2019 (1.3%), and they determined that SARS-CoV-2 may be blamed for BP in COVID-19 patients. In their 2021 retrospective analysis, Codeluppi et al. 23 noticed a higher prevalence of BP during the COVID-19 pandemic compared with the same period the previous year; according to them, 21% of BP patients presented active or recent SARS-CoV-2 infection symptoms, indicating a higher risk of developing BP during or after COVID-19.

Hogg et al. 24 found a substantial rise in the incidence of pediatric cases of BP during the COVID-19 epidemic compared with the previous year. Moreover, Brisca et al. 25 discovered an extraordinary rise in children with COVID-19-related FNP between March and April 2020.

According to Srinivas et al., 26 there were more cases of BP in 2020 (0.8%) than during the prepandemic period (0.05%). Moreover, Tamaki et al. 27 revealed a higher prevalence of BP in patients with COVID-19 in August 2021.

### Distribution of Patients according to Demographic Data

The present study included 27 patients with facial palsy with ages ranging from 2 to 16 (mean: 9.04 ± 3.14) years; only 2 (7.4%) had confirmed COVID-19 infection.

Theophanous et al. 2 reported the first pediatric case of BP associated with COVID-19 infection. They described a medically difficult case of a 6-year-old male patient with unilateral FNP and a positive SARS-CoV-2 RT-PCR. There was no history of trauma, illness, or recent travel.

On the other hand, a retrospective analysis of the prevalence of FNP between 2015 and 2020 was performed by Barron et al., 21 who found that the incidence of BP in 2020 did not differ from that of the previous 5 years. They could not find a connection between COVID-19 and FNP in children, as no evidence of COVID-19 infection was detected upon admission.

In a case series conducted in Brazil from May to July 2020, Lima et al. 12 identified 8 patients who had FNP during the pandemic and had positive SARS-CoV-2 RNA after reverse transcription-quantitative polymerase chain reaction (RT-qPCR) in nasal and nasopharyngeal swabs.

Likewise, Zammit et al. 22 reported a much higher incidence of FNP in 2020 than in 2019 and suggested the COVID-19 pandemic as the cause of the surge. Only 17 (57%) out of their 30 patients underwent COVID-19 testing, and among those, only 2 had a positive result.

### Change in House-Brackman Grade among the Studied Patients

One of the most frequently employed clinical evaluation methods is the H-B facial nerve grading system. The grades ranges from I (normal) to VI (severe), depending on the functional impairment (forehead movement, mouth asymmetry, movement, or eye closure) (no movement). We evaluated the H-B scale, which has been used consistently in the clinical practice to assess the severity of BP.

In the present study, in the assessments after one and three months, there was a significant improvement when compared with the assessment performed upon admission. After 6 months, the rate of grade-I patients was of 81.5%, with an insignificant change when compared with assessment after 3 months.

Yoo et al. 28 reported that presenting a lower initial H-B grade (II-IV) was strongly related to complete recovery, which they believed to be the most important factor associated with a favorable prognosis. This finding was similar to that of previous research in adults with BP, 38 which found that an initial lower degree of FNP, as measured by the H-B scale, was related to a better outcome. Yoo et al. 29 reported that BP patients had favorable outcomes at six months when the initial H-B grade was IV or lower. The facial nerve injury caused mild (grade II) impairment in 5 individuals and substantial (grade III) dysfunction in 3 patients, per the Lima et al. 12 study.

### Recovery from Facial Nerve Palsy

In pediatric patients, BP appears to recover earlier, and the overall recovery rate has been reported to be of up to 100%, which is higher than in adults. 17

In the present study, after 1 month, 15 patients (55.6%) presented complete improvement, while 3 patients (11.1%), no improvement, while in the third month, 20 patients (74.1%) were grade I, with significant improvement compared to the assessment after 1 month. After 6 months with insignificant change when compared with the assessment after 3 months, in which 22 patients (81.5%) showed complete improvement.

In their study, Lima et al. 12 found that, while 3 patients continued to present some degree of FNP at the last follow-up 30 days following the onset of neurological symptoms, 5 patients recovered completely. In the study by Gupta et al., 30 all seven children showed complete recovery.

Twelve case reports and one case series were included in the systematic review by Gupta et al., 31 and 16 out of the 20 individuals included had fully recovered from BP symptoms, whereas 4 had only partially recovered.

Egilmez et al. 32 reported that 31 cases had a history of contact with a COVID-19 patient, and 3 of those patients had positive COVID-19 RT-PCR results; they found a lower rate of full recovery: 37.5%.

### Relationship between Gender and Recovery from Facial Nerve Affection

In the present study, there was statistically insignificant relationship between age and recovery at the first, third, or sixth months. The mean age at the onset of BP was of 6.6 years in a study with 29 children conducted by Chen et al., 33 and of 9.2 years in a study with 106 children under the age of 15 performed by Jenke et al., 34 with no statistically significant difference between genders.

According to Lee et al., 35 age appears to be a good predictive factor for a quicker recovery from BP in children; they found no statistically significant correlation regarding prognosis and gender, the severity of the diagnosis, the affected side, and early or late treatment.

Hogg et al., 24 reported that the COVID-19 epidemic significantly increased the incidence of pediatric BP; their sample was composed of more female patients (12) than male patients (5).

Yoo et al. 28 concluded that a lower H-B grade at the initial presentation was the main factor impacting the full recovery from BP in children. Other factors, such as gender and age, failed to show any relevance in the prediction of the possibility of complete recovery.

Aslan et al. 36 found no evident change in terms of gender among their BP patients between the periods of January 2019 to January 2020 and April 2020 to April 2021.

### Relationship between COVID-19 Infection and Recovery from Facial Nerve Affection

The prognosis for BP in children is excellent; most children recover completely within 6 weeks, while some children may need up to 1 year. In general, the incidence of spontaneous recovery from BP in pediatric populations can reach 97%. 37

In a 2005 retrospective study, Chen et al. 33 reported a recovery rate of 68.8% after 3 weeks, and of 96.9% after 7 months, in a population of 29 children in Hong Kong. Moreover, in 2011, Jenke et al. 34 reported that, among 106 children in Germany, 97.6% recovered after 12 weeks, although only 3.7% of them had taken corticosteroids.

In the present study, there was a statistically insignificant relationship between COVID-19 infection and recovery in the first, third, or sixth months. According to the results, it seems that there is no association between COVID-19 infection and facial palsy; however, the patients infected with COVID-19 in the sample experienced a rapid, early recovery from BP. However, the present study is limited by the small sample size.

According to Mutlu et al., 20 98% of the patients who were referred to their clinic with idiopathic peripheral FNP received negative results from a SARS-CoV-2 RT-PCR test, and there was no rise in the number of patients with this condition during the first year of the COVID-19 pandemic. Moreover, Aslan et al. 36 concluded that they did not observe a relationship between COVID-19 and cases of BP.

Barron et al. 21 they examined many pediatric cases of FNP in a region of South Wales but were unable to establish a causal relationship between COVID-19 and pediatric FNP.

Evidence of BP in COVID-19 patients was found in the systematic review by Gupta et al., 31 suggesting a potential association with SARS-CoV-2;. 12 case reports and 1 case series serve as the foundation for this evidence. One of the shortcomings of their study 31 it is that they did not review a study with large sample size, a prospective study, or a randomized controlled study.

Moreover, Codeluppi et al. 23 reported that there were more cases of facial palsy during the first year of the COVID-19 pandemic than there had been during the same time the year before, suggesting an elevated risk of FNP during or following COVID-19 infection. The fundamental weakness in their study 23 is that, despite the fact that 21% of the sample from the 2020 cohort displayed COVID-19 clinical symptoms, 37 out of the 38 included patients were not tested for SARS-CoV-2. This is why it is incomprehensible that the review and conclusion used just clinical data and excluded the findings of PCR tests.

Codeluppi et al. 23 should have assessed if there was a link between COVID-19 and FNP in the 1 COVID-positive patient with facial palsy in their study. The results of nerve conduction studies (NCSs), brain imaging findings, and cerebrospinal fluid (CSF) findings would be of particular interest. Before attributing an increased frequency of facial palsy to the infection, it is critical to establish a causal relationship between COVID-19 and BP. Furthermore, according to Codeluppi et al., 23 in 2019 there were 2 patients with COVID-19-associated FNP. However, the outbreak had not yet reached Europe at that point. How do the authors address this contradiction?

The main limitation of the present study was the small sample size; larger cohort studies and multicenter studies are required to support our findings. Another limitation to the study is the period of follow-up of facial nerve recovery. We followed up our patients for up to 6 months. A longer period of follow-up may be required for patients without recovery of facial nerve function in up to one year.

## Conclusion

In the present study, we were not able to show any association between BP and COVID-19. We also followed up with the patients for up to 6 months, in addition to studying their patterns of recovery, which were similar to those observed before the pandemic.
